# Dietary glycaemic index, glycaemic load and head and neck cancer risk: a pooled analysis in an international consortium

**DOI:** 10.1038/s41416-019-0702-4

**Published:** 2020-01-13

**Authors:** Chun-Pin Chang, Carlo La Vecchia, Diego Serraino, Andrew F. Olshan, Jose P. Zevallos, Hal Morgenstern, Fabio Levi, Werner Garavello, Karl Kelsey, Michael McClean, Chu Chen, Stephen M. Schwartz, Stimson Schantz, Guo-Pei Yu, Paolo Boffetta, Mia Hashibe, Yuan-Chin Amy Lee, Maria Parpinel, Livia S. A. Augustin, Federica Turati, Zuo-Feng Zhang, Valeria Edefonti

**Affiliations:** 1 Division of Public Health, Department of Family & Preventive Medicine, University of Utah School of Medicine, and Huntsman Cancer Institute, 375 Chipeta Way, Suite A, Salt Lake City, UT 84108 USA; 2 Department of Clinical Sciences and Community Health, Università degli Studi di Milano, via Venezian, 1, 20133 Milano, Italy; 3 Cancer Epidemiology Unit, Centro di Riferimento Oncologico di Aviano (CRO) IRCCS, via Gallini, 2, 33081 Aviano, Italy; 4 Department of Epidemiology, Gillings School of Global Public Health, Campus Box 7435, Chapel Hill, NC 27599 USA; 5 Division of Head and Neck Surgical Oncology, Department of Otolaryngology/Head and Neck Surgery, Washington University School of Medicine, 660 S. Euclid Ave., Campus Box 8115, St. Louis, MO 63110 USA; 6 Departments of Epidemiology and Environmental Health Sciences, School of Public Health, and Department of Urology, Medical School, University of Michigan, 1415 Washington Heights, M5164 SPH-II, Ann Arbor, MI 48109-2029 USA; 7 Institut Universitaire de Médecine Sociale et Préventive (IUMSP), Unisanté, University of Lausanne, Route de la Corniche 10, 1010 Lausanne, Switzerland; 8 Department of Otorhinolaryngology, School of Medicine and Surgery, University of Milano-Bicocca, via Pergolesi, 33, 20052 Monza, Italy; 9 Department of Epidemiology and Department of Laboratory Medicine and Pathology, Brown University, 70 Ship Street, Providence, RI 02912 USA; 10 Boston University School of Public Health, 715 Albany St Talbot Building, Boston, MA 02118 USA; 11 Program in Epidemiology, Division of Public Health Sciences, Fred Hutchinson Cancer Research Center, 1100 Fairview Avenue North, Seattle, WA 98109 USA; 12 New York Eye and Ear Infirmary, 310 East 14th Street, New York, NY 10003 USA; 13 Department of Otolaryngology, School of Medicine, New York Medical College, Valhalla, New York, NY USA; 14 The Tisch Cancer Institute, Mount Sinai School of Medicine, 17 E 102 St 4th Floor Room 4-110, New York, NY 10029 USA; 15 Department of Medical and Surgical Sciences, University of Bologna, via Massarenti, 9, 40138 Bologna, Italy; 16 Department of Medicine, University of Udine, via Colugna, 50, 33100 Udine, Italy; 17 Epidemiology and Biostatistics Unit, Istituto Nazionale Tumori IRCCS “Fondazione G. Pascale”, via Semmola, 53, 80131 Napoli, Italy; 18 Clinical Nutrition and Risk Factor Modification Centre, St. Michael’s Hospital, 61, Queen St. East, Toronto, ON M5C 2T2 Canada; 19 Department of Epidemiology, UCLA Fielding School of Public Health, 71-225 CHS, Box 951772, Los Angeles, CA 90095 USA

**Keywords:** Head and neck cancer, Cancer epidemiology, Risk factors, Cancer prevention, Epidemiology

## Abstract

High dietary glycaemic index (GI) and glycaemic load (GL) may increase cancer risk. However, limited information was available on GI and/or GL and head and neck cancer (HNC) risk. We conducted a pooled analysis on 8 case-control studies (4081 HNC cases; 7407 controls) from the International Head and Neck Cancer Epidemiology (INHANCE) consortium. We estimated the odds ratios (ORs) and 95% confidence intervals (CIs) of HNC, and its subsites, from fixed- or mixed-effects logistic models including centre-specific quartiles of GI or GL. GI, but not GL, had a weak positive association with HNC (OR_Q4 vs. Q1_ = 1.16; 95% CI = 1.02–1.31). In subsites, we found a positive association between GI and laryngeal cancer (OR_Q4 vs. Q1_ = 1.60; 95% CI = 1.30–1.96) and an inverse association between GL and oropharyngeal cancer (OR_Q4 vs. Q1_ = 0.78; 95% CI = 0.63–0.97). This pooled analysis indicates a modest positive association between GI and HNC, mainly driven by laryngeal cancer.

## Background

Most head and neck cancers (HNCs) are attributed to tobacco smoking and/or alcohol drinking.^1^ Diet has been suggested to play a role in HNC aetiology, with non-starchy vegetables and selected healthy dietary patterns being inversely related with HNC risk.^2^

Average daily glycaemic index (GI) ranks carbohydrate foods based on the postprandial blood glucose response; average glycaemic load (GL) estimates the impact of carbohydrate consumption using the GI, while taking into account the amount of carbohydrates that are consumed.^3^ Higher GI and GL are moderately associated with risk of several cancers,^4^ likely because of stimulation of insulin release and bioactivity of insulin-like growth factor-1, which has proliferative, angiogenic, anti-apoptotic and oestrogen stimulating properties.^5^

Only two studies^6,7^ have investigated the effect of GI and GL on HNC risk, with inconsistent findings; one of these studies^6^ reported results by sub-site, based, however, on a limited number of cases.

The objective of this paper is to assess the association of GI or GL with HNC and its subsites (i.e. oral cavity, oropharynx, hypopharynx and larynx) using pooled dietary data from eight case-control studies participating in the International Head and Neck Cancer Epidemiology (INHANCE) consortium.^8^

## Methods

Within data version 1.5 of the INHANCE dataset, information on GI and GL was available from 3 case-control studies. In addition, we calculated GI and/or GL intakes from study-specific food items and food composition databases for another five studies, giving a total of eight studies included in the analysis. Details on individual studies and data pooling methods have been previously described^8^ and are summarised in Supplementary Table S1. Informed consents and institutional review board approvals were obtained within the framework of the original studies.

### Selection of subjects

Cases were included if their cancer had been originally classified as invasive cancer of the oral cavity, pharynx, larynx or unspecified oral cavity/pharynx. Corresponding controls from the original studies were included in the analysis. We excluded subjects with missing information on the site of origin of cancer, or GI or GL value, and those with missing or implausible (<500 or >5500 kcal/day) non-alcohol energy intake. Thus, our analysis included 11,488 subjects, with 4081 HNC cases and 7407 controls (4264 hospital-based and 3143 population-based controls). There were 810 oral cavity, 1172 oropharynx, 343 hypopharynx, 1338 larynx and 418 unspecified oral cavity/pharynx cancer cases.

### Specification of variables

Study-specific food-frequency questionnaires (FFQs) and food composition tables allowed us to calculate individual values of GI and GL for the four studies lacking information on both the exposures [Los Angeles, Boston, Seattle (1985–1995) and Memorial Sloan Kettering Cancer Center (MSKCC) studies]. In detail, as described previously,^9^ the GI of a food was expressed as a percentage of the glycaemic response elicited by white bread as a standard food with a GI of 100. The average daily GI for each subject was computed by summing the products of the GI value of each food times the amount of available carbohydrates in that food consumed per day, divided by the total amount of available carbohydrates (g) consumed per day. The average daily GL (g) was calculated by summing the products of the GI value of each food times the amount of available carbohydrates in that food consumed per day, divided by 100. Each GL unit represents the equivalent of 1 g of carbohydrate from white bread. Therefore, we initially converted frequencies of consumption into servings/day and servings/day into grams/day; then, we assigned the corresponding GI to each food item and applied the previous formulas to derive individual GI and GL values. For the North Carolina (2002–2006) study, information on individual values of GL was originally provided to the INHANCE Consortium Coordinating Center. We estimated GI as 100 multiplied with GL and divided by total available grams of carbohydrate intake (Supplementary Material—text and Table S2 for GI/GL calculation and study-specific GI values).

### Statistical analysis

Multiple logistic regression models were used to estimate the odds ratios (ORs) of HNC and the corresponding 95% confidence intervals (CIs) according to centre-specific quartiles of GI or GL among controls (Supplementary Tables S3 for descriptive statistics of GI and GL distributions). In the presence of heterogeneity of GI or GL intakes across centres, we used a random-slope logistic regression model, whereas a fixed-effects model was used otherwise.^10^ The models included the following potential confounders: age, sex, race/ethnicity, study centre, education, cigarette smoking intensity, cigarette smoking duration, cigar smoking status, pipe smoking status, alcohol drinking intensity and the product term of cigarette smoking and alcohol drinking intensities. For GI, models were further adjusted for energy intake without alcohol; for GL, models were further adjusted for energy intake without alcohol and carbohydrates. For both GI and GL models, we used centre-specific control-based quartiles of energy intake. Separate analyses were carried out by HNC subsites and in strata of selected covariates. In sensitivity analyses, we further adjusted for history of diabetes or excluded subjects with diabetes (information available for 6 studies). Analyses were performed using the SAS software (version 9.4, SAS Institute, Cary, NC).

## Results

Characteristics of our sample were presented in Supplementary Table S4. The highest GI quartile category (Q4) was associated with a higher HNC risk (OR_Q4 vs. Q1_ = 1.16; 95% CI = 1.02–1.31, p_trend_ = 0.037, Table 1). Across HNC subsites, GI was associated with an increased laryngeal cancer risk (OR_Q4 vs. Q1_ = 1.60; 95% CI = 1.30–1.96, p_trend_ < 0.001), but excluding laryngeal cancer cases, the OR_Q4 vs. Q1_ was 1.01 (95% CI = 0.88–1.16, p_trend_ = 0.90) (data not shown). Little associations between GL and cancers of the oral cavity, hypopharynx and larynx were observed. An inverse association was found between oropharyngeal cancer risk and GL (OR_Q4 vs. Q1_ = 0.78; 95% CI = 0.63–0.97, p_trend_ = 0.009). Results did not materially change when excluding subjects with diabetes or when additionally adjusting models by diabetes history. No heterogeneity was observed in strata of covariates (Supplementary Table S5).

**Table 1:** Odds ratios (ORs)^a^ and 95% confidence intervals (CIs) of glycaemic index and glycaemic load on cancers of head and neck, oral cavity, oropharynx, hypopharynx and larynx.

	Head and neck cancer (No. cases = 3967, No. controls = 7250^b^)	Oral cavity cancer (No. cases = 780, No. controls = 7250^b^)	Oropharyngeal cancer (No. cases = 1151, No. controls = 7250^b^)	Hypopharyngeal cancer (No. cases = 328, No. controls = 6866^b^)	Laryngeal cancer (No. cases = 11,299, No. controls = 6443^b^)
	OR (95% CI)	OR (95% CI)	OR (95% CI)	OR (95% CI)	OR (95% CI)
Glycaemic index					
I Quartile	Reference	Reference	Reference	Reference	Reference
II Quartile	1.04 (0.91, 1.18)	0.92 (0.70, 1.20)	0.93 (0.77, 1.13)	0.87 (0.61, 1.23)	1.33 (1.08, 1.65)
III Quartile	1.02 (0.90, 1.17)	1.08 (0.66, 1.75)	0.90 (0.74, 1.09)	0.95 (0.68, 1.35)	1.28 (1.04, 1.59)
IV Quartile	1.16 (1.02, 1.31)	1.21 (0.81, 1.81)	0.93 (0.76, 1.12)	0.84 (0.59, 1.20)	1.60 (1.30, 1.96)
P_for linear trend_	0.037	0.63	0.40	0.46	<0.001
P_heterogeneity_^c,d^	0.35	0.03	0.41	0.22	0.66
Glycaemic load					
I Quartile	Reference	Reference	Reference	Reference	Reference
II Quartile	0.94 (0.82, 1.07)	0.88 (0.70, 1.12)	0.91 (0.75, 1.11)	0.78 (0.54, 1.13)	0.95 (0.76, 1.18)
III Quartile	0.89 (0.78, 1.02)	0.86 (0.68, 1.10)	0.75 (0.61, 0.92)	0.74 (0.51, 1.09)	1.03 (0.83, 1.28)
IV Quartile	0.91 (0.79, 1.05)	0.89 (0.69, 1.15)	0.78 (0.63, 0.97)	0.84 (0.57, 1.22)	1.03 (0.82, 1.28)
P_for linear trend_	0.15	0.37	0.009	0.41	0.63
P_heterogeneity_^c,d^	0.52	0.30	0.97	0.24	0.68

International Head and Neck Cancer Epidemiology (INHANCE) consortium

^a^Models adjusted for age, sex, race/ethnicity, study center, education level, center-specific control-based quartiles of energy intake (without alcohol for glycaemic index; without alcohol and carbohydrate for glycaemic load), cigarette smoking intensity (number of cigarettes per day), cigarette smoking duration, cigar smoking status, pipe smoking status, alcohol drinking intensity (number of drinks per day) and the product (interaction) term for cigarette smoking intensity and alcohol drinking intensity

^b^The number of controls differed across subsites because a few studies considered cancers of the oral cavity, oropharynx and hypopharynx only; therefore, they contributed to the analysis with fewer controls than those studies with all cancer subsites included (see Supplementary Table 4)

^c^P-value for heterogeneity between study centers

^d^Based on the likelihood ratio test of heterogeneity between study centers, we reported the fixed-effects estimates when P_heterogeneity_  >  0.1 and the mixed-effects estimates when P_studies_  <  0.1

## Discussion

In this large dataset, we observed a positive association between GI and HNC risk, essentially driven by laryngeal cancer. GL was not associated with the risk of overall HNC or its subsites, except for a possible inverse association with oropharyngeal cancer.

Inconsistent associations of GI and GL with HNC risk may be partly due to differences in the underlying dietary patterns. Indeed, higher dietary GL is strongly associated with higher carbohydrate intakes, while a higher GI is also associated with lower intakes of dairy products, legumes, fruit and vegetables.^11^ In line with this hypothesis, an overlapping INHANCE-based analysis including seven of the eight current studies showed a positive association of laryngeal cancer with an “*Animal products and cereals”* dietary pattern, which was simultaneously based on high-GI (e.g. cereals) and low-GL (e.g. meat) foods.^10^

Only two previous studies^6,7^ have examined the association between GI or GL and HNC risk, with one of them partially overlapping with the current dataset.^6^ An analysis^6^ of three Italian case-control studies on upper aero-digestive tract cancers reported a positive association with higher GI (OR_Q5 vs. Q1_ = 1.5; 95% CI = 1.1–2.0) and GL (OR_Q5 vs. Q1_ = 1.8; 95% CI = 1.1–2.9) in quintiles. Although in the same direction, the association was weaker with oral and pharyngeal cancers combined or laryngeal cancer.^6^ Findings from the National Institutes of Health–AARP Diet and Health Study (1239 HNC cases; 446,177 participants) reported a null association with GI and a possible inverse association with GL in women (OR_Q5 vs. Q1_ = 0.63; 95% CI = 0.34–1.19), in the absence of a clear dose-response relationship.^7^

Limitations of the current analyses included possible recall bias and non-differential misclassification of GI/GL quartiles. In addition, food items contributing to GI differed in part across regions (Supplementary Table S2). However, all our FFQs were either reproducible and valid or were modifications of existing FFQs, already tested for reproducibility and validity. We were able to adjust for major potential confounders and our large sample size provided the necessary statistical power to examine the association in HNC subsites and strata.^8^

In conclusion, findings from this large-scale pooled analysis support a positive effect of average daily GI on the risk of HNC, and in particular of laryngeal cancer.

## Supplementary information

Supplementary Material

## Data Availability

The dataset used and analysed during the current study is available from the corresponding author on reasonable request.
