# Supplementary material for: Dietary glycaemic index, glycaemic load and head and neck cancer risk: a pooled analysis in an international consortium
Source: Br J Cancer. 2020 Jan 13;122(6):745–8. doi: 10.1038/s41416-019-0702-4 (PMC7078183; doi:10.1038/s41416-019-0702-4)
Supplement: Supplementary file 1 — Supplementary Material [file 41416_2019_702_MOESM1_ESM.pdf]

Supplementary Material

Manuscript: "Dietary glycemic index, glycemic load, and head and neck cancer risk: a pooled analysis in an international consortium"

METHODS (extended)

Glycemic index and glycemic load checking

Information on individual values of glycemic index (GI) and glycemic load (GL) was originally provided to the International Head and Neck Cancer Epidemiology (INHANCE) Consortium Coordinating Center by the Principal Investigators of the Italy Multicenter, Switzerland, and Milan (2006-2009) studies. Calculation of GI and GL was based on the same (reproducible and valid) food-frequency questionnaire (FFQ) and food-composition tables[1] across the three studies. An extended description was provided in full elsewhere.[2] For those studies, we checked missing or inconsistent values and solved inconsistencies, when possible.

Glycemic index and glycemic load estimation

GI and GL were estimated for Los Angeles, Boston, Seattle (1985-1995) and Memorial Sloan Kettering Cancer Center (MSKCC) studies from their FFQs by the following steps:

1. Converting the consumption frequency to servings per day

For Boston and Seattle (1985-1995) studies, a daily serving for each food item was obtained by DIETSYS Nutrient Analysis System. For the Los Angeles study, daily serving was calculated by using consumption frequency times the weight of none, daily, weekly, monthly and yearly consumption frequency (0, 1, 1/7, 1/30.42 and 1/365, respectively). For the MSCKK study, the raw data from INHANCE consortium only contained the information of frequency per month instead of daily, weekly, monthly and yearly consumption frequency. Hence, a daily serving was calculated by:

(monthly frequency/30.42) * the serving size. The weights for small, medium and large serving size were 0.5, 1 and 1.25, respectively.

2. Converting daily serving to daily intake in gram

Grams per portion size for each food item were obtained from the U.S. Department of Agriculture (USDA).[3] USDA Nutrient Database Standard Reference, version 16 (SR16)[4] provides the grams per portion size as well as the nutritional composition for each food item. Daily intake (gram) was calculated by multiplying the daily serving of specific food item by its grams per portion size. Available carbohydrate per 100 grams for each food item was also obtained from the same source. We defined available carbohydrate to be the USDA-based value for grams of carbohydrate per 100 grams minus the USDA value for grams of dietary fiber per 100 grams. Daily available carbohydrate intake (g/day) was calculated by summing the products of available carbohydrate (g/100g) of the specific food item by daily intakes (g) and dividing by 100.

3. Assigned glycemic index value to each food item

We linked GI values (using a scale assuming bread=100) to each food item using the published GI estimates. We searched for the most similar food item within the international GI tables[5,6], considering only studies in healthy subjects and conducted in the United States or Canada. Whenever more than one GI value was provided for the same type of food in the international table, the average GI value was assigned to that food item. When the food item could not be found in the tables, we then searched the GI values compiled by Flood et al..[7] The process of linkage was carried

out by manual reviewing the GI tables to identify the best matches for each food item in the questionnaire.

4. Daily glycemic index and glycemic load calculation

Average dietary GI and GL were calculated by the following formula[3,8,9]:

$$Average\ dietary\ GI = \frac{\sum(GI\ \ of\ each\ food\ item * available\ grams\ of\ carbohydrate\ intake\ of\ each\ food\ item)}{total\ available\ grams\ of\ carbohydrate\ intake}$$

$$Daily\ GL = \frac{\sum(GI\ \ of\ each\ food\ item * available\ grams\ of\ carbohydrate\ intake\ of\ each\ food\ item)}{100}$$

where the sum was carried out across all foods consumed by each subject. Each GL unit represents the effect of consuming one gram of carbohydrate from bread.

For the North Carolina (2002-2006) study, information on individual values of daily GL was originally provided to the INHANCE Consortium Coordinating Center by the Principal Investigators. We estimated dietary GI as 100 multiplied with GL divided by total available grams of carbohydrate intake.

Supplementary Tables

Supplementary Table 1. Characteristics of individual studies in the International Head and Neck Cancer Epidemiology (INHANCE) Consortium used in the current analysis.

Study Reference paper	Recruitment period	Source (cases/ controls)	Participation rate, % (cases/controls)	Age eligibility (years)	Number of subjects (cases/ controls)	Questionnaire, administration, reference period for the recall, reproducibility and validity	Frequency	Serving size[a]	# Food items (including non-alcoholic beverages)
**Italy Multicenter** Bosetti et al., 2003[b]	1990-1999	Hospital/Hospital-unhealthy	>95/>95	18-80	1261/2716	FFQ, interviewer-administered, 2 year before disease, reproducible and valid	Raw data	S/M/L	78 (including 6 non-alcoholic beverages)
**Switzerland** Levi et al., 1998[b]	1991-1997	Hospital/Hospital-unhealthy	>95/>95	<80	516/883	FFQ, interviewer-administered, 2 year before disease, reproducible and valid	Raw data	S/M/L	78 (including 6 non-alcoholic beverages)
**Los Angeles**, CA, USA Cui et al., 2006	1999-2004	Cancer registry/ Neighborhood	49/68	18-65	417/1005	FFQ, interviewer-administered, during the past year, modification of an existing FFQ, tested for reproducibility and validity	Raw data	M	78 (including 11 non-alcoholic beverages)
**Boston**, MA, USA Peters et al., 2005	1999-2004	Hospital/ Residential records	88.7/48.7	≥18	584/659	FFQ, self-administered, during the past year, reproducible and valid	Categories	M	138 (including 12 non-alcoholic beverages)
**New York, MSKCC**, USA Schantz et al., 1997	1992-1994	Hospital/Blood donors	NA	NA	134/169	FFQ–diet history, self-administered, during the past year, modification of an existing FFQ, tested for reproducibility and validity [c]	Raw data	S/M/L	88 (including 5 non-alcoholic beverages)
**Milan (2006-2009)**, Italy Bravi et al., 2013 [b]	2006-2009	Hospital/Hospital-unhealthy	>95/>95	18-80	367/750	FFQ, interviewer-administered, 2 years before disease, reproducible and valid	Raw data	S/M/L	78 (including 6 non-alcoholic beverages)
**North Carolina (2002-2006)**, USA Divaris et al., 2010[c]	2002-2006	Cancer registry/ DMV files	82/61	20-80	1368/1396	FFQ, interviewer-administered, during the past year, modification of an existing FFQ, tested for reproducibility and validity	Categories	M	72 (including 5 non-alcoholic beverages) questions
**Seattle (1985-1995)**, WA, USA Rosenblatt et al, 2004 [d]	1985-1995	Cancer registry/ Random digit dialing	54.4/63.3; 63.0/60.9	18-65	407/607	FFQ, interviewer-administered, 5 years ago, reproducible and valid	Raw data	S/M/L	106 (including 7 non-alcoholic beverages)

ABBREVIATIONS: DMV: Department of Motor Vehicles; FFQ: food-frequency questionnaire; S: small; M: medium; MSKCC: Memorial Sloan Kettering Cancer Center; L: large; NA: not available.

a. A quantification of the medium serving size was provided in all the studies. b. Italy Multicenter, Milan (2006-2009) and Switzerland studies were based on the same food-frequency questionnaire. c. The food-frequency questionnaire from the North Carolina study provided combined questions concerning consumption of specific food items and corresponding condiment habits or fat content of the food item of interest (i.e. while asking for cooked or raw vegetable consumption, the food frequency questionnaire asked for extra information on fat, sauce, or dressing added after cooking or at the table). d. Two response rates are reported because data were collected in two population-based case-control studies, the first from 1985 to 1989 among men and the second from 1990 to 1995 among men and women.

Supplementary Table 2. Glycemic index values for selected food items in the food frequency questionnaires included in the current analysis. International Head and Neck Cancer Epidemiology (INHANCE) consortium.

Boston and North Carolina (2002-2006)[a]			Los Angeles			MSKCC and Seattle[a]			Italy Multicenter, Switzerland, and Milan (2006 - 2009)[a]		
**Food item**	**GI**	**GI x CHO[b]**	**Food item**	**GI**	**GI x CHO[b]**	**Food item**	**GI**	**GI x CHO[b]**	**Food item**	**GI**	**GI x CHO[b]**
Potatoes-bake/boil/mash	158	3865	Other white potatoes (boiled, baked, potato salad, mashed)	159	3762	Other potatoes, yams	158	1932	Maize (polenta)	106	4325
Pretzels	119	2564	Other cold cereals such as Corn Flakes, Rice Krispies	114	2847	Other cold cereals	114	2840	Bread	101	3232
Cold breakfast cereal	114	2136	Corn bread, corn muffins, corn tortillas	108	2920	Watermelon (in season)	109	592	Biscuits	95	4057
Pancakes/waffles[c]	110	5578	White bread (including sandwiches), bagels, etc.	104	6075	Other soups	108	1721	Fruit or jam pies	93	6092
Chowder/cream soup	108	1721	Watermelon (in season)	103	1118	Corn bread, corn muffins, corn tortillas	108	2924	Sugar	89	262
Regular muffins/biscuits	107	3327	Rice	103	3985	White bread, rolls, crackers (include sandwiches)	105	4198	Pizza	86	6321
Doughnuts	107	2682	Cantaloupe (in season)	93	1079	Rice	103	3389	Risotto	86	6125
White bread, including pita	105	2099	French fries, fried potatoes, hash browns	91	2764	Other fruit juices, fortified fruit drinks	97	2324	Ice cream	83	1718
Jam/jelly/syrup/honey	104	1404	Regular soft drinks (not diet)	90	6298	Hamburgers, cheeseburgers, meat loaf	94	3000	Lasagne, tortellini with meat filling	64	3611
White rice	103	4519	Beef stew or pot pie with vegetables	89	1093	Cantaloupe (in season)	93	299	Pasta/rice with tomato sauce	62	4371

ABBREVIATIONS: GI: glycemic index; CHO: available carbohydrate; MSKCC: Memorial Sloan Kettering Cancer Center.

a. For Italy Multicenter, Switzerland, and Milan (2006 - 2009) studies, the same questionnaire was used. For Boston, North Carolina, MSKCC, and Seattle studies, different questionnaires with similar high glycemic index food items were used. We, therefore, combined their information in this table.

b. GI x CHO: product of the (available) carbohydrates content per food serving times its GI value in the food-frequency questionnaire.

c. Pancakes/waffles item was present in the Boston study only.

Supplementary Table 3. Descriptive statistics on glycemic index and glycemic load across study centers and in all the studies combined. International Head and Neck Cancer Epidemiology (INHANCE) consortium.

Study Center	Q1 (25th)	Median	Q3 (75th)
**Glycemic Index**			
Overall	71.53	76.25	81.07
Boston	79.69	82.74	85.5
Italy Multicenter			
Pordenone	70.61	74.77	78.7
Milan	70.88	74.56	78.06
Latina	72.29	76.19	79.31
Los Angeles	72.37	77.33	82.52
MSKCC	76	80.16	83.97
Milan (2006-2009)	74.64	78.44	81.55
North Carolina (2002-2006)	69.55	72.97	76.39
Seattle (1985-1995)	76.96	80.02	83.64
Switzerland	67.9	75.01	82.94
**Glycemic Load**			
Overall	141.69	191.73	253.07
Boston	169.39	221.98	284.74
Italy Multicenter			
Pordenone	171.7	213.63	267.14
Milan	146.54	191.72	240.18
Latina	165.07	213.3	260.19
Los Angeles	113.74	161.49	238.64
MSKCC	90.42	138.24	182.26
Milan (2006-2009)	167.86	203.88	250.75
North Carolina (2002-2006)	119.86	163.59	217.67
Seattle (1985-1995)	119.75	166.65	207.98
Switzerland	112.37	169.94	245.69

ABBREVIATIONS: MSKCC: Memorial Sloan Kettering Cancer Center.

Supplementary Table 4. Distribution of selected characteristics among controls and cancer cases of head and neck, oral cavity, oropharynx, hypopharynx, and larynx. International Head and Neck Cancer Epidemiology (INHANCE) consortium.

	Controls (7407), n(%)	Head and neck cases (4081), n(%)	Oral cavity cases (810), n(%)	Oropharynx cases (1172), n(%)	Hypopharynx cases (343), n(%)	Larynx cases (1338), n(%)
**Age (years)**						
17 to 40	461 (6.2)	152 (3.7)	41 (5.1)	46 (3.9)	2 (0.6)	22 (1.6)
40 to 44	434 (5.9)	195 (4.8)	41 (5.1)	63 (5.4)	13 (3.8)	46 (3.4)
45 to 49	720 (9.7)	475 (11.6)	93 (11.5)	177 (15.1)	36 (10.5)	111 (8.3)
50 to 54	1206 (16.3)	668 (16.4)	130 (16.0)	234 (20.0)	67 (19.5)	170 (12.7)
55 to 59	1338 (18.1)	823 (20.2)	161 (19.9)	258 (22.0)	75 (21.9)	257 (19.2)
60 to 64	1084 (14.6)	638 (15.6)	123 (15.2)	159 (13.6)	60 (17.5)	242 (18.1)
65 to 69	1021 (13.8)	569 (13.9)	105 (13.0)	139 (11.9)	52 (15.2)	231 (17.3)
70 to 74	847 (11.4)	391 (9.6)	74 (9.1)	76 (6.5)	28 (8.2)	186 (13.9)
75 to 89	294 (4.0)	170 (4.2)	42 (5.2)	20 (1.7)	10 (2.9)	73 (5.5)
Missing	2 (0.0)	0 (0.0)	0 (0.0)	0 (0.0)	0 (0.0)	0 (0.0)
**Sex**						
Female	2382 (32.2)	874 (21.4)	269 (33.2)	217 (18.5)	57 (16.6)	196 (14.6)
Male	5020 (67.8)	3202 (78.5)	538 (66.4)	954 (81.4)	286 (83.4)	1141 (85.3)
Missing	5 (0.1)	5 (0.1)	3 (0.4)	1 (0.1)	0 (0.0)	1 (0.1)
**Race**						
Black	340 (4.6)	335 (8.2)	62 (7.7)	81 (6.9)	23 (6.7)	118 (8.8)
Others (with Asians)	107 (1.4)	76 (1.9)	9 (1.1)	29 (2.5)	3 (0.9)	15 (1.1)
White (with Hispanics)	6917 (93.4)	3653 (89.5)	733 (90.5)	1060 (90.4)	315 (91.8)	1200 (89.7)
Missing	43 (0.6)	17 (0.4)	6 (0.7)	2 (0.2)	2 (0.6)	5 (0.4)
**Study center**						
Boston	611 (8.2)	358 (8.8)	76 (9.4)	160 (13.7)	33 (9.6)	64 (4.8)
Italy Multicenter						
Milan	621 (8.4)	193 (4.7)	57 (7.0)	57 (4.9)	24 (7.0)	24 (1.8)
Pordenone	1527 (20.6)	880 (21.6)	79 (9.8)	220 (18.8)	105 (30.6)	436 (32.6)
Latina	425 (5.7)	95 (2.3)	40 (4.9)	34 (2.9)	7 (2.0)	0 (0.0)
Los Angeles	1018 (13.7)	400 (9.8)	49 (6.0)	144 (12.3)	16 (4.7)	83 (6.2)
MSKCC	123 (1.7)	106 (2.6)	52 (6.4)	11 (0.9)	10 (2.9)	32 (2.4)
Milan (2006-2009)	691 (9.3)	331 (8.1)	79 (9.8)	36 (3.1)	14 (4.1)	203 (15.2)
North Carolina (2002-2006)	1120 (15.1)	1057 (25.9)	152 (18.8)	298 (25.4)	47 (13.7)	373 (27.9)
Seattle (1985-1995)	394 (5.3)	175 (4.3)	93 (11.5)	74 (6.3)	0 (0.0)	0 (0.0)
Switzerland	877 (11.8)	486 (11.9)	133 (16.4)	138 (11.8)	87 (25.4)	123 (9.2)
**Education**						
No education	20 (0.3)	14 (0.3)	3 (0.4)	4 (0.3)	1 (0.3)	5 (0.4)
Less than junior high school	2603 (35.1)	1386 (34.0)	221 (27.3)	332 (28.3)	143 (41.7)	592 (44.2)
Some high school	803 (10.8)	678 (16.6)	162 (20.0)	185 (15.8)	62 (18.1)	215 (16.1)
High school graduate	917 (12.4)	660 (16.2)	133 (16.4)	185 (15.8)	58 (16.9)	197 (14.7)
Technical school, some college	1510 (20.4)	748 (18.3)	176 (21.7)	239 (20.4)	44 (12.8)	204 (15.2)
More than college graduate	1550 (20.9)	588 (14.4)	114 (14.1)	224 (19.1)	35 (10.2)	123 (9.2)
Missing	4 (0.1)	7 (0.2)	1 (0.1)	3 (0.3)	0 (0.0)	2 (0.1)

ABBREVIATIONS: MSKCC: Memorial Sloan Kettering Cancer Center.

Supplementary Table 4. (continued) Distribution of selected characteristics among controls and cancer cases of head and neck, oral cavity, oropharynx, hypopharynx, and larynx. International Head and Neck Cancer Epidemiology (INHANCE) consortium.

	Controls (7407), n(%)	Head and neck cases (4081), n(%)	Oral cavity cases (810), n(%)	Oropharynx cases (1172), n(%)	Hypopharynx cases (343), n(%)	Larynx cases (1338), n(%)
**Cigarette smoking status**						
Never	3111 (42.0)	578 (14.2)	137 (16.9)	216 (18.4)	24 (7.0)	80 (6.0)
Former	3060 (41.3)	1738 (42.6)	244 (30.1)	523 (44.6)	150 (43.7)	683 (51.0)
Current	1210 (16.3)	1749 (42.9)	424 (52.3)	430 (36.7)	169 (49.3)	566 (42.3)
Missing	26 (0.4)	16 (0.4)	5 (0.6)	3 (0.3)	0 (0.0)	9 (0.7)
**Cigarette smoking intensity (number of cigarettes/day)**						
Never smokers	3111 (42.0)	579 (14.2)	137 (16.9)	216 (18.4)	24 (7.0)	81 (6.1)
> 0 to 10	1367 (18.5)	460 (11.3)	85 (10.5)	150 (12.8)	29 (8.5)	138 (10.3)
> 10 to 20	1791 (24.2)	1493 (36.6)	285 (35.2)	403 (34.4)	124 (36.2)	563 (42.1)
> 20 to 30	530 (7.2)	719 (17.6)	137 (16.9)	198 (16.9)	71 (20.7)	250 (18.7)
>30 to 40	352 (4.8)	558 (13.7)	110 (13.6)	145 (12.4)	61 (17.8)	204 (15.2)
40+	184 (2.5)	227 (5.6)	45 (5.6)	53 (4.5)	24 (7.0)	88 (6.6)
Missing	72 (1.0)	45 (1.1)	11 (1.4)	7 (0.6)	10 (2.9)	14 (1.0)
**Cigarette smoking duration (years)**						
Never smokers	3111 (42.0)	579 (14.2)	137 (16.9)	216 (18.4)	24 (7.0)	81 (6.1)
> 0 to 10	560 (7.6)	143 (3.5)	25 (3.1)	62 (5.3)	6 (1.7)	20 (1.5)
> 10 to 20	858 (11.6)	263 (6.4)	62 (7.7)	83 (7.1)	20 (5.8)	67 (5.0)
> 20 to 30	1062 (14.3)	691 (16.9)	139 (17.2)	222 (18.9)	69 (20.1)	203 (15.2)
>30 to 40	978 (13.2)	1173 (28.7)	242 (29.9)	339 (28.9)	114 (33.2)	398 (29.7)
40+	820 (11.1)	1220 (29.9)	200 (24.7)	247 (21.1)	110 (32.1)	565 (42.2)
Missing	18 (0.2)	12 (0.3)	5 (0.6)	3 (0.3)		4 (0.3)
**Cigar smoking status**						
Never cigar user	6980 (94.2)	3729 (91.4)	747 (92.2)	1073 (91.6)	318 (92.7)	1219 (91.1)
Ever smoked more than 100	402 (5.4)	330 (8.1)	56 (6.9)	95 (8.1)	22 (6.4)	112 (8.4)
Missing	25 (0.3)	22 (0.5)	7 (0.9)	4 (0.3)	3 (0.9)	7 (0.5)
**Pipe smoking status**						
Never pipe user	6870 (92.8)	3747 (91.8)	742 (91.6)	1073 (91.6)	316 (92.1)	1235 (92.3)
Ever smoked more than 100	508 (6.9)	311 (7.6)	64 (7.9)	97 (8.3)	24 (7.0)	90 (6.7)
Missing	29 (0.4)	23 (0.6)	4 (0.5)	2 (0.2)	3 (0.9)	13 (1.0)
**Alcohol drinking intensity (number of drinks/day)**						
Never drinker	1741 (23.5)	395 (9.7)	91 (11.2)	107 (9.1)	16 (4.7)	114 (8.5)
<1	2260 (30.5)	674 (16.5)	154 (19.0)	225 (19.2)	18 (5.2)	172 (12.9)
1 to < 3	1898 (25.6)	797 (19.5)	158 (19.5)	233 (19.9)	57 (16.6)	279 (20.9)
3 to < 5	818 (11.0)	607 (14.9)	125 (15.4)	146 (12.5)	59 (17.2)	222 (16.6)
5+	689 (9.3)	1605 (39.3)	281 (34.7)	461 (39.3)	193 (56.3)	550 (41.1)
Missing	1 (0.0)	3 (0.1)	1 (0.1)	0 (0.0)	0 (0.0)	1 (0.1)

ABBREVIATIONS: MSKCC: Memorial Sloan Kettering Cancer Center.

Supplementary Table 5. Odds ratios (ORs)[a] and 95% confidence intervals (CIs) of glycemic index and glycemic load on head and neck cancer in strata of selected covariates. International Head and Neck Cancer Epidemiology (INHANCE) consortium.

	Glycemic Index				Glycemic Load			
	II Quartile	III Quartile	IV Quartile	$P_{studies}$[c]	II Quartile	III Quartile	IV Quartile	$P_{studies}$[c]
**Age (years)**								
17 to 54	1.20 (1.00, 1.45)	1.15 (0.97, 1.36)	1.34 (1.10, 1.64)	0.923	1.06 (0.82, 1.37)	1.03 (0.77, 1.37)	0.99 (0.76, 1.30)	0.805
55 to 89	0.93 (0.76, 1.14)	0.98 (0.71, 1.35)	1.06 (0.83, 1.36)	0.058	1.00 (0.77, 1.31)	0.89 (0.62, 1.28)	0.95 (0.74, 1.23)	0.023
**P_{strata}[b]**		0.676				0.690		
**Sex**								
Female	1.04 (0.81, 1.33)	1.08 (0.84, 1.40)	1.23 (0.96, 1.58)	0.051	0.92 (0.72, 1.18)	0.99 (0.76, 1.28)	1.02 (0.76, 1.37)	0.550
Male	1.04 (0.89, 1.21)	1.00 (0.86, 1.16)	1.11 (0.96, 1.29)	0.565	0.93 (0.79, 1.09)	0.85 (0.72, 1.00)	0.87 (0.74, 1.02)	0.598
**P_{strata}[b]**		0.277				0.677		
**Education**								
<= Junior high school	0.65 (0.37, 1.15)	0.77 (0.54, 1.11)	0.92 (0.53, 1.58)	0.061	0.90 (0.64, 1.26)	0.78 (0.53, 1.14)	0.64 (0.34, 1.17)	0.003
<= High school	1.10 (0.81, 1.51)	1.12 (0.77, 1.62)	1.53 (1.04, 2.26)	0.331	1.11 (0.67, 1.84)	0.95 (0.60, 1.51)	1.20 (0.73, 1.96)	0.272
>= Some college	1.02 (0.83, 1.25)	1.05 (0.89, 1.26)	1.07 (0.91, 1.27)	0.504	1.09 (0.92, 1.29)	0.95 (0.84, 1.08)	0.90 (0.77, 1.04)	0.970
**P_{strata}[b]**		0.991				0.516		
**Tobacco smoking status**								
Never	0.91 (0.70, 1.19)	1.02 (0.78, 1.32)	1.03 (0.79, 1.34)	0.572	1.06 (0.81, 1.38)	0.93 (0.70, 1.24)	0.89 (0.65, 1.22)	0.328
Former	1.11 (0.92, 1.34)	1.03 (0.85, 1.25)	1.24 (1.03, 1.49)	0.579	0.97 (0.80, 1.19)	0.92 (0.75, 1.12)	0.98 (0.80, 1.21)	0.708
Current	1.01 (0.77, 1.31)	0.99 (0.76, 1.28)	1.10 (0.86, 1.42)	0.356	0.82 (0.63, 1.07)	0.86 (0.66, 1.14)	0.82 (0.62, 1.08)	0.634
**P_{strata}[b]**		0.621				0.855		
**Cigarette smoking intensity (number of cigarettes/day)**								
Never	0.91 (0.70, 1.19)	1.02 (0.79, 1.33)	1.03 (0.79, 1.34)	0.573	1.07 (0.82, 1.39)	0.93 (0.70, 1.24)	0.89 (0.65, 1.22)	0.312
>0 to 20	1.10 (0.92, 1.33)	0.99 (0.82, 1.19)	1.25 (1.04, 1.50)	0.246	0.93 (0.77, 1.12)	0.85 (0.70, 1.03)	0.96 (0.79, 1.18)	0.700
>20	0.98 (0.75, 1.28)	1.08 (0.83, 1.40)	1.10 (0.85, 1.42)	0.526	0.90 (0.69, 1.19)	1.00 (0.76, 1.32)	0.81 (0.61, 1.08)	0.465
**P_{strata}[b]**		0.830				0.533		
**Alcohol drinking intensity**								
Never/light	1.08 (0.88, 1.34)	1.03 (0.83, 1.28)	1.16 (0.95, 1.43)	0.414	1.14 (0.93, 1.41)	1.00 (0.79, 1.25)	1.04 (0.82, 1.32)	0.779
Moderate	1.04 (0.84, 1.29)	1.07 (0.87, 1.32)	1.14 (0.92, 1.41)	0.272	0.89 (0.72, 1.10)	0.85 (0.68, 1.06)	0.90 (0.71, 1.14)	0.495
Heavy	1.03 (0.77, 1.38)	1.11 (0.83, 1.49)	1.27 (0.96, 1.69)	0.943	0.75 (0.54, 1.04)	0.86 (0.62, 1.18)	0.79 (0.57, 1.08)	0.988
**P_{strata}[b]**		0.623				0.063		
**BMI (kg/m^2)**								
Underweight	0.50 (0.18, 1.40)	1.12 (0.50, 2.51)	1.09 (0.25, 4.73)	0.310	0.50 (0.16, 1.54)	0.75 (0.23, 2.37)	0.62 (0.16, 2.36)	0.294
Normal	1.04 (0.84, 1.30)	1.13 (0.91, 1.41)	1.22 (1.00, 1.48)	0.630	0.90 (0.72, 1.12)	0.79 (0.63, 1.00)	0.81 (0.64, 1.02)	0.737
Overweight/Obese	1.07 (0.77, 1.50)	0.95 (0.56, 1.60)	1.02 (0.78, 1.32)	0.014	0.97 (0.81, 1.16)	0.94 (0.78, 1.13)	0.95 (0.78, 1.15)	0.153
**P_{strata}[b]**		0.471				0.472		
**Study design**								
Hospital-based controls	1.20 (1.00, 1.45)	1.13 (0.94, 1.36)	1.29 (1.07, 1.54)	0.238	0.96 (0.79, 1.16)	0.95 (0.78, 1.15)	1.00 (0.82, 1.22)	0.261
Population-based controls	0.90 (0.74, 1.08)	0.94 (0.79, 1.13)	1.08 (0.91, 1.30)	0.561	0.98 (0.81, 1.18)	0.89 (0.73, 1.09)	0.84 (0.68, 1.04)	0.911
**P_{strata}[b]**		0.983				0.672		
**Region**								
Europe	1.20 (1.00, 1.45)	1.10 (0.91, 1.32)	1.29 (1.07, 1.55)	0.384	0.93 (0.77, 1.13)	0.92 (0.76, 1.12)	0.98 (0.81, 1.20)	0.431
North America	0.90 (0.75, 1.09)	0.98 (0.82, 1.17)	1.09 (0.91, 1.30)	0.388	1.01 (0.84, 1.22)	0.93 (0.77, 1.13)	0.88 (0.71, 1.08)	0.554
**P_{strata}[d]**		0.770				0.432		

a. Adjusted for age, sex, race/ethnicity, study center, education levels, energy intake (without alcohol for glycemic index; without alcohol and carbohydrate for glycemic load), cigarette smoking intensity (number of cigarettes per day), cigarette smoking duration, cigar smoking status, pipe smoking status, alcohol drinking intensity (number of drinks per day), and the product (interaction) term for cigarette smoking and alcohol drinking, when appropriate. b. P for heterogeneity across strata. c. P for

heterogeneity between study centers. When $P_{studies} < 0.1$ in one of the strata, we consistently reported the stratum-specific mixed-effects estimates for every stratum.

References

1. Salvini S, Parpinel M, Gnagnarella P, et al. (1998) Banca Dati di composizione degli alimenti per studi epidemiologici in Italia. Milano, Italy: Istituto Europeo di Oncologia.
2. Augustin LS, Dal Maso L, La Vecchia C, Parpinel M, Negri E, Vaccarella S *et al.* Dietary glycemic index and glycemic load, and breast cancer risk: a case-control study. *Annals of oncology : official journal of the European Society for Medical Oncology* 2001; **12**(11): 1533-1538.
3. Liu S, Willett WC, Stampfer MJ, Hu FB, Franz M, Sampson L *et al.* A prospective study of dietary glycemic load, carbohydrate intake, and risk of coronary heart disease in US women. *The American journal of clinical nutrition* 2000; **71**(6): 1455-1461.
4. U.S. Department of Agriculture ARS. USDA National Nutrient Database for Standard Reference, Release 16-1. In. Nutrient Data Laboratory Home Page, /nuteintdata2004.
5. Atkinson FS, Foster-Powell K, Brand-Miller JC. International tables of glycemic index and glycemic load values: 2008. *Diabetes care* 2008; **31**(12): 2281-2283.
6. Foster-Powell K, Holt SH, Brand-Miller JC. International table of glycemic index and glycemic load values: 2002. *The American journal of clinical nutrition* 2002; **76**(1): 5-56.
7. Flood A, Subar AF, Hull SG, Zimmerman TP, Jenkins DJ, Schatzkin A. Methodology for adding glycemic load values to the National Cancer Institute Diet History Questionnaire database. *Journal of the American Dietetic Association* 2006; **106**(3): 393-402.
8. Michaud DS, Fuchs CS, Liu S, Willett WC, Colditz GA, Giovannucci E. Dietary glycemic load, carbohydrate, sugar, and colorectal cancer risk in men and women. *Cancer epidemiology, biomarkers & prevention : a publication of the American Association for Cancer Research, cosponsored by the American Society of Preventive Oncology* 2005; **14**(1): 138-147.
9. Wolever TM, Jenkins DJ, Jenkins AL, Josse RG. The glycemic index: methodology and clinical implications. *The American journal of clinical nutrition* (Review) 1991; **54**(5): 846-854.
